# Mistakes and Failures in Bridge-Work and Some Remedies

**Published:** 1907-05-15

**Authors:** Frank E. Logan


### MISTAKES AND FAILURES IN BRIDGE-WORK AND SOME REMEDIES.

BY FRANK E. LOGAN, D.D.S.

Read before the Detroit Dental Society, March 14, 1907.

When reading the articles published in our dental journals, I have often thought that if some of the many eminent authors instead of elaborating their uninterrupted successes, would for a change relate a few of their failures, they might prove as instructive in the end, and they would surely make many a poor and discouraged practitioner feel that he was not alone in his miserable failures. But it is universally the rule that we hear of nothing but an endless chain of successes with no links of failures interposed here and there to weaken it.

Failures in my opinion are responsible for many successes in life, for nobody ever achieved the highest ideal without failing some times. Failures should be made forerunners of success. Each failure should teach the avoidance of that particular danger in the future, for when everything goes along smoothly and you seem on the high road to success, that is the most dangerous time. When humbled by the lessons of disappointment, we are in better spirit to win success. To the energetic and ambitious practitioner adversity will prove to be only a temporary delay, for it will make him the more determined to attain a higher degree of accomplishment along the particular lines of failure.

From my own view-point, I consider a failure a personal humiliation; but we all have them, and it should be our highest ambition to make a success in future out of each of them. You all appreciate the genuine satisfaction there is in "winning out" after you have failed repeatedly on some troublesome case. So don't give up until you have succeeded in making good in every case you undertake.

In my opinion if bridge work, whether fixed or removeable, is honestly recommended, properly and skillfully executed on thoroughly prepared anchorages, so that when completed it presents an artistic and esthetic appearance, you need have little or no fear of failure; for in such a piece of work you have the most comfortable and useful of all artificial substitutes and no other method of procedure seems to offer so great an oppurtunity for the servicable, and I may say, the permanent reproduction of the normal condition. But why so many failures?

Let us start at the very begining. In recent years there has been considerable controversy regarding "Higher Standards in Dental Education" and "Errors in Dental Education," in many of our magazines, and invariably the substance of these articles has been, that the student about to enter college, has not always received the proper preliminary education to fit him for the work he is about to enter upon. He should have had more chemistry, physics, biology, etc., at High School. Granting the truth of the assertion and that he has been justly criticized for his shortcomings by the professors in our institutions. May I in behalf of the student turn the "lime light" on some of our instructors in crown and bridge work, who pride themselves on being specialists in this partic ular branch.

Have these instructors and demonstrators been appointed to the positions they occupy because they possess the scientific and techical ability to fill the position with credit to themselves and honor to the college they represent? Have they endeavored to make the very best use of their opportunities to advance themselves in this special department of dentistry that they may be properly equipped to demonstrate intelligently to the student the most improved and up to date methods in connection with this exceedingly intricate work? If they have not done so, let these instructors examine themselves, let the analysis be thorough and when they have finished, they may have found that failures in this work do not necessarily originate with the student, but may perhaps be due to the faulty instructions he has received.

Again it is not a recognized fact that many of our demonstrators are graduates of the college in which they are teaching and as a consequence their views along this special work are sure to be narrow. Perhaps they have received their appointments through some personal or political pull, or they may be giving their time to the institution gratis in order to be associated with it as members of the faculty, that they may gain prestage with the public in the City in which they practice.

If the conditions I have just cited exist in our colleges, and I have very good reasons for believing that they do, is it to be wondered at that our graduates are badly handicapped in their training along certain lines, and as a result that they and their patients have many failures to contend with, on account of the lack of technical instruction they should have received.

There is no doubt in my mind that more than 90 per cent of all failures originate in the faulty preparation of the teeth that are to serve as abutments. It is entirely unnecessary for me to enter into details regarding the manner a tooth should be treated that is to be utilized for the support of a bridge, for there is not a practitioner present who does not know when he has done the work conscientiously and if he feels that he does not have the time or possess the required patience and ability to reduce it to the proper shape, he had better let bridge-work alone and insert plates.

It is absolutely essential that the crown should fit perfectly at the gingival margin, otherwise the work cannot be expected to be permanent. The care with which the abutments have been prepared, and I cannot emphasize too strongy the importance of using the utmost care and patience in this part of the work.

The preparation of the tooth for an abutment is one of the most important features in successful bridge-work and nothing short of perfection will give us the results we hope to obtain.

The tooth must be trimmed so that when the measurement is taken the circumference is greatest just below the gingival margin; considerable skill is required to accomplish this desired shape more especially in the molar region, if the adjacent tooth is in place, for it makes the disto-lingual angle difficult to prepare on account of the limited space. However, with Leaming and diamond disks kept wet by a spray of water, you can soon reduce the bulge of tooth substance so that the root's largest circumference is at the gum line. A tooth prepared in this manner, is almost impervious to any external agency entering when the crown has been accurately adjusted, but should the preparation be done in a slovenly haphazard manner, the crown fails to fit closely at the neck of the tooth, and as a result the cement will dissolve, decomposing food particles and other conditions favoring decay will lodge in the pocket previously filled by the cement and very soon the entire tooth is destroyed by decay, sometimes without any warning to the patient.

Bridge-work will surely fail when placed on weak or unhealthy roots, or when the tissue surrounding the roots is diseased. There is absolutely no reason for using a root in such condition for an abutment, and such procedure is nothing short of criminal, for no matter how perfectly a bridge may have been constructed from the mechanical standpoint, from the practical you can expect nothing but a failure.

Nearly all diseased roots are affected by pyorrhoea. If the abscess is chronic and cannot be thoroughly eradicated by therapeutic treatment, surgical procedure should be resorted to and the apex of the root amputated, and should the root be necrosed, every particle of the necrotic tissue should be thoroughly removed and a radical cure produced, otherwise the diseased tooth should be extracted.

If the necrosed root be on one of the multi-rooted teeth, the sacrifice of the entire tooth is seldom necessary, for if this particular tooth is to form the posterior abutment for a bridge and there is no tooth beyond it, and you resort to extraction, you have mutilated your case to such an extent that a fixed bridge cannot be inserted and have also made the conditions more difficult to remedy if you contemplate inserting a removeable one.

Let us suppose the bicuspid teeth and the second and third molars on one side of the superior maxillary have been extracted, but the first molar is still in position with the palatine root badly necrosed, and owing to the pus organisms suppuration has completely destroyed the vitality of the pericementum, and absorption of the alveolus has been so pronounced that there is no longer any attachment to the surrounding tissue; in other words, the root is absolutely dead. Are we justified in extracting such a tooth? If so, do you think that as capable surgeons, we have done our patient the best possible service or have we done them an irreparable injury? I maintain that such procedure is less excusable than if a general surgeon should insist on removing the entire hand because a portion of the bone in the little finger is necrosed.

In the case cited the entire palatine root should be amputated, for we cannot hope to correct such a pathological condition by medical treatment, and when this is accomplished suppuration will invariably cease almost immediately, and the remaining roots will become firm in their sockets and retain the tooth rigidly in the process, and regain a usefulness that is nothing short of miraculous in the eyes of the patient. What a fatal mistake it would have been to have removed such a tooth, for when the overhanging walls are trimmed down so as to parallel the remaining roots you will have a posterior support for your bridges that will give you very gratifying results.

Experience has taught us that the most successful bridges have been small ones, which have been attached to strong healthy roots or teeth. Bridges of ten or more are seldom admissible under any circumstances, unless the abutments are so placed as to equalize the strain or pressure caused by the force of mastication.

A bridge is a rigid structure and when it is fixed to two or more supports, it can only assume the movement of one. The larger and more extensive the bridge, the more complicated become the forces that act upon it, Nature is generous in this direction, however, but there are cases when it sees fit to relieve itself of burdensome intrusions. There are certain prescribed limits wherein we are allowed to operate, and as soon as we understand the principles and laws that should govern us within these limits, and the failures and disasters that await us without, we shall have made some progress in the development of this most interesting work.

One of the greatest mistakes made to day by many practitioners is the indiscriminate and unskillful use of open-faced crowns and bands as attachments for bridge-work. Personally I claim they are a curse to the success of bridge-work, and such pernicious procedure cannot be too strongly condemned. I honestly believe that from both practical experience and observation more good, strong healthy teeth have been sacrificed and more failures resulted from the use ot these abominable open-taced crowns than trom any other method ot attachment; and yet you will hear dentists elaborate upon the merits ot this torm ot device, claiming that you are not obliged to mutilate the tooth in order to apply it. I can not agree with them, tor all the anterior teeth are so shaped that it is practically impossible to band them pertectly at the gingival margin without grinding the tooth so as to uttely destroy its appearance, tor as previously stated, a tooth prepared to receive a crown must have its greatest circumierence at the gum line.

No matter how thoroughy or accurately the band has been adapted, the tooth substance is liable to disintigrate in a very few years especially if the tissue is lacking in lime salts and consequently is weak, and as a result you will have nothing to show but a failure for a piece of work that was supposed to be permanent.

Where the open-faced crowns are employed, there occurs in many instances a complete obliteration of the inter dental space, the gold comes in direct contact with the approximal surface of the adjoining tooth and forms an admirable lodging place for debris, and in a very short time a cavity develops in this surface, a condition which would not likely have occured had a proper attachment been used. I firmly believe that these open faced crowns are contraindicated in every tooth in the mouth excepting the lower cuspids and centrals, the former being usually cone shaped and requiring little or no shaping at all to receive the band, and the latter on account of preserving the natural crowns of the teeth, in order to give greater strength to the attachment, for these lower centrals are small and delicate in their structure, and should the natural crowns be incised it tends to weaken the attachment by lessening the strength of the abutment.

From the esthetic standpoint these bands and crowns are decidedly inartistic, they are injurious and often destructive to the tooth to which they are attached, and are also a menace to the permanancy of the bridge because they constitute its weakest part.

I am a firm believer in "Extension for Prevention" in bridge-work as in operative work and when I can see that by incising the natural crown of a tooth and applying a Richmond crown for an abutment I can procure a greater degree of permanancy a more artistic effect, and more satisfactory results to my patient, I do not hesitate to do so, I fear that many of us are too conservative regarding this method of procedure, for when the root of the incised tooth is healthy and thoroughly filled at the apex and a properly constructed Richmond crown applied, you have one of the most perfect abutments for the anterior teeth that could possibly be inserted for a fixed bridge. It is strong, artistic and cleanly. There is absolutely no possibility of solution of cement and subsequent loosing of the piece, as is liable to occur where a band or jacket crown is attached.

The beauty of all artificial work lies in the ability of the artist to conceal his art, and no where is this more applicable than in crown and bridge work. Do you think that bands, open-faced crowns and gold crowns in the front of a patient's mouth assist in disguising it?

Suffice to say, that if dentistry is to become universally acknowledged as a profession, embracing a field of dignified and scientific pursuit, and if crown and bridge-work is ever to be accorded that recognition of an art to which the scope of its possibilities entitles it, the practice of placing gold bands and gold crowns on any of the six anterior teeth or within the range of vision, violates every principle of art. It is degrading and cannot be too vigorously condemned, for no matter how well the work has been performed, it is an offense to art and refinement.

A number of failures in bridge-work have resulted where the operator aimed to make a permanent piece of work, by anchoring its adjoining teeth by means of fillings either gold or amalgam or gold inlays. It is not surprising that such work is unsuccessful, considering that the principle is entirely at variance with the results desired. It is impossible to suppose that an occluso-proximal cavity of fairly good proportions can support the strain of mastication that is exerted on two or even one additional tooth, when experience teaches us that fillings in similar positions and of like dimensions frequently break down by the force exerted upon them. For in the molar region as you are all well aware, there is a strain of from 70 to 250 pounds per individual tooth. Furthermore, by virtue of the elasticity of the alivola process and the joint peculiar to the natural tooth, there is always a certain amount of movement to be overcome, and the security this mode of attachment offers, is as a rule far from sufficient, either the filling becomes loosened or the bar becomes detached. None of us would think of filling two opposing approximal cavities in adjoining permanent teeth, with one filling and expect a successful nseration, yet the principal is identical. This class of work is not practicable and seldom permissible and cannot be expected to be successful, excepting in a case where the occlusion is very slight or wanting.

Considerable trouble has arisen from crowning and bridging teeth containing live pulps. The feasibility of sacrificing the pulps in teeth that are to be subsequently used for the abutment of a bridge is of the utmost importance to both the operator and patient. This subject has occasioned much discussion in the profession, and many practitioners, such as Peeso, Harlan, Kirk, Rhein and others favor the devitalization, while other men of good judgment such as Black, Guilford and Hunt denounce such procedure as not being conducive to the best and most permanent results.

When a tooth has been crowned an abnormal condition has been established, and if the vitality of the pulp has been preserved, it is subjected to deleterious influences, sometimes caused by the action of the acid in the cement, but this can be remedied to a great extent by cauterizing the tooth thoroughly with silver nitrate, which is self limiting in its action and will by coagulating the albumen in the tubuli overcome the irritating influence and also prevent to a great extent the influence of thermal changes.

We encounter a more serious problem when we endeavor to overcome the excessive irritation that often follows the preparation of a tooth caused by cutting and grinding. Such procedure is very liable to cause a marked inflammation of the pulp, and unless relieved, will result in congestion which finally terminates in the death of the pulp, and this way be followed by disagreeable pathological conditions, such as pericementitis and eventually the formation of an alveolar abscess.

If the preparation of a tooth can be accomplished without any undue mechanical irritation or discomfort to the patient, the proposed bridge not being an extensive one, I believe in preserving the vitality of the pulp, but when the piece is extensive and I feel that there is the slightest possibility of any pathological disturbance resulting in the future, I do not hesitate to resort to a prevention procedure in every instance and devitalize. To accomplish this I prefer the use of cocaine to arsenic, for I believe the latter is responsible many times for inflammatory conditions that develop later and may terminate in peridental or alveolar disease.

It has been conceded by some of the best authorities in dentistry that the embryologic if not the physiologic function of the pulp is discontinued with the complete development of the teeth, which is usually about the eighteenth or twentieth year, so that after the maturity of the tooth, the pulp is not absolutely necessary for its stability. Accordingly a great deal is gained by destroying it whenever we have any doubts concerning its vitality, thus saving ourselves considerable trouble and the patient untold suffering at some future time. This mode of treatment is not advisable in mouths of patients under eighteen years of age, for it is questionable if the teeth are fully developed before that period. However, little or no bridge-work is necessary at that age, unless it is to assist the Orthodontists in supplying unerupted teeth. Nor is it necessary to destroy all pulps in the teeth of adults who have reached the age of forty or fifty because of the general tendency of atrophy of the organ, which together with the secondary deposit of dentin materially decreases the sensitiveness of the tooth so that the preparation can be accomplished with scarcely any irritation, consequently there is little danger of any future complications presenting themselves.

Faulty articulation has been responsible for numerous failures. It will play havoc with what might have been a perfect piece of work. The occlusion with the teeth, in the opposing jaw has everything to do with the success of bridge-work and nothing short of perfection in this particular will answer.

Bridges of sufficient solidity, with abutments equally as firm are often capable of resisting direct force or pressure caused by malocclusion but will be utterly destroyed by indirect impacts. The most destructive of all these forces is the lateral thrust as where the jaw is moved forward or sideways against the bridge as a lever. In such cases the strain of mastication will very soon force the abutments out of line and cause marked irritation of the membrane and we are obliged to add one more failure to our list.

Discouraging failures have resulted from the unskillful use of the blow pipe and the utter disregard of certain requirements that must be observed if we wish to eliminate the danger of accidents, such as burning the case, checking the porcelain facings, and balling up of solder.

It is necessary that all the parts to be soldered should be absolutely clean before such procedure is ahttempted, any wax on the tooth should be removed by placing in chloroform; the metal parts should be treated to an acid bath to remove all oxidation and base metal, and thorough apposition of the cusps and backing should be observed. When backing the tooth allow the material to project beyond the approximal sides so that when the teeth are assembled in the bridge and ready for soldering, the backing will lap upon each other, thus avoiding those troublesome joints between the facing as well as the objectionable V-shaped spaces.

In investing the case, use as little material as is practicable to hold the parts firmly in position. I do not know of a more admirable substance for this work than that used by Dr. Peeso of Philadelphia. It is ordinary red bird gravel or sand, two parts to one of plaster which gives a dense and strong investment, capable of being carved or trimmed to a very compact form and showing no preceptible shrinkage when heated.

When ready for soldering, place the case over a burner on a disk of copper plate, so as to diffuse the heat, making sure that the porcelain facings are heated first, as you are all well aware, the porcelain being a mineral substance absorbs the heat more slowly than the platinum pins and consequently the expansion of this substance should precede that of the pins, otherwise a fractured facing is liable to result. Therefore be careful in applying the heat to see that it is gradual and uniform, dust the surface to be soldered very lightly with dry powdered borax and increase the temperature of the piece with the blow pipe until it reaches a red glow. Now you are ready for soldering the solder having previously been cut into a long strip, dip in liquid flux and apply to the particular part of the bridge you wish to unite, at the same time cutting down the flame so that it will play directly on the extremity of the solder, unite the cusp to the abutments first and then the cusps to each other and by so doing you will tend to overcome shrinkage by avoiding an excess of solder in any one area. When this has been done, keep feeding your solder in at the lingual edge of the cusps until the desired contour has been acquired. By manipulating the solder in this manner, you can direct it to the exact spot you wish it to flow, there is no "balling up" and no air bubbles are confined, causing a porous piece when completed, and if the slightest care is exercised it is next to impossible to burn the bridge.

Do not throw your solder into the case like so much kindling in a wood box and expect that after you have given it a generous sprinkling of borax and heated it to a temperature sufficiently high to cause it to fuse that you will not have your troubles. It is almost sure to ball up and in your endeavor to make it flow after it has been thoroughly oxidized, the first thing you know you have alloyed the gold in your bridge and a burned case is the result. If the dentist will apply himself closely to a few rules that must be observed, he will soon acquire such skill as will render this procedure one of the simplest in dentistry and he will be surprised with what ease and dexterity successful results can be accomplished.

In concluding I have no hesitancy in saying that there is no form of replacement that can approach in appearance, comfort and utility that of a well conceived, proportioned and constructed bridge. I may also add with propriety, that another accompaniment to a finished bridge is cleanliness. This property of cleanliness is what appeals to me so strongly in favoring removable bridge-work, for from the hygenic, practicable and mechanical standpoint it discounts fixed bridge-work in every respect.

Such bridges can be easily removed by the patient, thoroughly sterilized and readily replaced, insuring a healthy condition of the membranes of the mouth and a clean sweet breath.

Another very strong point in its favor is that in event of an accident, a removable bridge is so easily repaired, as the work can be done in the laboratory without any loss of time or discomfort to the patient.

Should an abutment loosen, it can be reset in a few minutes; to remedy such a condition in a fixed bridge of several teeth, sometimes necessitates the ruination of the entire piece, especially if a couple of Richmond crowns are involved.

All things being considered, there is no question that a removable bridge is the most perfect and excellent form of replacement; and to be successful in this work a man should be a thinker, must possess keen judgment and discernment, for so many complicated cases arise that each one requires a special study. He should be an accurate and skillful mechanic, a metallurgist and above all an "artist."

### DISCUSSION.

DR. WARD: While in general I agree with the paper, there are a few things with which naturally you would expect some one would disagree. There are no two of us that would work always alike in order to accomplish exactly the same thing.

The essayist says that our failures are not mentioned. It is natural that a man does not like to mention his failures to be criticized. I do not tell mine and the other fellow does not tell his.

The point brought up in regard to "the lime-light on our instructors." I agree that it is a good thing to do and it is good to turn it on every one of us. When it is turned upon us justly it does us all good. A good smooth road and easy time will make a fizzle out of almost any of us.

The point brought out in regard to the roots not being properly prepared is timely. I think most of us have put in bridge-work in which we could have prepared the roots better; we did not have the time, did not take the time, or did not know how. When we get back to the second molar to prepare it to the size it ought to be at the gingiva, it is a hard thing to do and many of us stop short of accomplishing what we ought to do. It will take you a couple of hours, as a rule, to prepare it as it ought to be prepared.

I cannot accomplish the preparation of a second or first molar stump, or even a bicuspids, with discs. I had called to my attention this fall a little cone shaped fissure burr which comes to a point. It is made by Ivory, and cuts well, with this in angle hand pieces. You can reduce the size of the stump until the greatest circumference is just under the gum line. I do not know of anything better in the line of burrs than this conical shaped cross-cut fissure burr. For the anterior roots a set of cleavers devised by Dr. Wells of Minnesota, now being made by the Cleveland Dental are excellent. They are, I should think, about $\frac{1}{3}$ the size of the Case Cleavers and can be slipped under the gum of the anterior teeth very nicely. If they are used judiciously I think that we can get the circumference of the stump down to somewhere near where it ought to be. I cannot accomplish the same ends with any disc.

The essayist maintains that bridge-work is a failure if placed on weak or unhealthy roots, or when the surrounding tissue is diseased. This apparently condemns the application of bridge-work to an important class of abutments, but later he mentions amputating the lingual roots of an upper first molar. We know that if there is any great movement at all it is bucco-lingually. If we have a patient who has pyorrhoea and we should have to take out some part of the roots to save the tooth we have to anchor the bridge-work to what we have left of the tooth. If the roots are then made solid so much the better, but if they are bad or loose we must use them anyway. If the tissues surrounding are diseased, I think that in most cases they can be treated so that the bridge may be a success. We can usually take care of that part of it. I agree with the essayist that the small bridges are the most successful and that those larger ones are permissible only when the abutments are well distributed.

He stated that the open faced crowns are a mistake. I think his statement is about right but I take exception to his conclusion, and think his rule is entirely wrong. As a general rule bands that are placed upon teeth are a menace but who of you ever saw a band that was put around a tooth that was not all right while it was tight. If a plate is soldered to the lingual side of a cuspid it will offer more surface for retention and retain its position for a long time. These bands are not, as a rule, made strong enough and the cement loosens and secretions get under them. A great many dentists whom I know are making a shoe for the lingual surface and are making successful work, and that is in a good many ways an open face crown. The band well fitted to some of the teeth, not letting it come to the labial, but covering the mesial and distal and also the lingual, can be used to good advantage.

So far as disintegration of the live tooth is concerned, the percentage of lime salts has nothing to do with this disintegration. The open faced crown, the essayist says is indicated only on the lower cuspids and incissors. I think it is indicated also on the upper cuspids and incisors. There we are often called upon to supply a bridge. I do not apply the open face crown in the sense that a good many apply a so called slipper, I know of a great many skillful men who are called upon to do something of this kind, and they agree that it is a good work if well done.

I disagree just as heartily with the essayist in regard to gold and amalgam fillings and gold inlays and think as a result of their insertion bridge-work is often a failure. Now if we had the case of a youngster of the age of 15 or 16 that had been to the orthodontist and previous to that had had his first molars out below. If the orthodontist had restored the space of the first molar and sent him back to you, what, would you put in? Would you crown the same second bicuspid or second molar or both? Would you put a gold inlay in one of them and put a lug in? Yes. Would you put it in both of them? Maybe you would. You certainly would not devitalize and crown at the age of 17 a tooth that had not a cavity in it just because a gold inlay or amalgam filling was not strong enough. I know a man who has to do work inexpensively but wants to do a good class of work. He has at the posterior and anterior end of his bridge a spur or knob, which he is anchoring into the distal of a bicuspid and the mesial side of the molar. He may cut freely out the mesial of the molar to take out the pulp. These anchorages will stand if put well over into the occlusal surface far enough and fastened into the alloy fillings.

On the other hand, suppose we have the first bicuspid gone in the upper jaw, and we still have the second bicuspid and the cuspid, both good strong teeth, would you cut them both off and crown them? I think you would better use a gold inlay or other filling for attachment.

Some reference was made to the value of the pulp in bridge work. The essayist quoted some authors to say that it had no function after the tooth had developed, and others to show that it had.

How may a tooth deteriorate in strength, with one who has no bad habits, at what age is it strongest, and what is the controlling factor in the strength of the teeth?

In answer to these questions I would say that anything which tends to lessen the organic material in the teeth weakens the tooth. We have learned that the composition of the teeth is approximately 10 per cent of water, 65 per cent of inorganic matter, and 25 per cent of organic matter; that at the age of 25 or 30 the organic matter is the greatest and at that age the teeth are the strongest. As the person gets older these lime salts increase and instead of the teeth getting stronger they become more brittle and weaker. The function of the pulp is to supply the organic material through the contents to the tubuli. If then the pulp is of no value in itself we might say that indirectly it adds strength because it supplies the organic material through the tubuli to nourish and strengthen the tooth. I cannot therefore agree to the devitalization of pulps.

We have used everything in the category of medicines to control the bad tendency of arsenic to make the peridental tissues sore, without definite results. We can sever the nerve easily at the apex in some roots but not in the first upper bicuspids, or any of the molars. I usually leave the pulp chamber open a week or ten days with a proper dressing and wait for it to slough down. We are in the habit of combining arsenic with morphine and cocaine and essential oils, or carbolic acid. I have nursed a few cases in which some essential oil excipient had been used. These oils are the most penetrating substance which you could use arsenic with, and should not be applied in any case. In my hands carbolic acid has proven the most successful excipient and I have never had a case that has given me trouble when carbolic acid was used. The thing that has troubled me much is cases where you have injected the pulp with high pressure. In many cases the roots of an upper molar, will some of them be lame for two or three days afterwards. I have injected three teeth on the same day and removed the pulps from all of them, and only one got lame, but it remained so three or four days. That is one of the troubles I have had. The other day I took the pulp out of a molar tooth in which one root was not anesthetized at all while the lingual root got enough cocaine to make the gum as far as the palate numb. I think the best way is to put into the nerve paste something that will break down the pulp and disintegrate it entirely. I agree with the essayist that the articulation of bridge teeth should be given much more careful attention than has ever been given to this part of the work.

In the use of the blow pipe and application of solder there is one thing I wish to mention. When we flow solder we forget that pure platinum or gold backings are soluble in hot metals. We say we have burned our backing, gold or platinum, still we have not approached the degree of volatization at all, nor oxidation; we have simply put the backings into solution in the solder. Solder just as quickly as you can and pull the flame off, at the fusing point, if you persist for a second too long part of the backing goes into solution.

Another thing I would caution you against is not to apply large quantities of borax to a case that required absolutely none. If you have backed your facings with pure gold or platinum, they are not affected by the heat, but you should keep the borax off of them. If you have a 22 carat abutment, put on a little dry borax, and apply the solder in a long strip, heat the case where the solder will flow and by holding it above the inverted bridge with small pliers, then fuse it as quickly as you can. Be careful not to keep the solder liquid long nor put on large quantities of borax.

While we all desire good and sanitary bridges and can use removable work in some cases, the time is not quite yet when we can say that we shall practice all removable work, or all fixed work. I think that there are cases where we must use our best judgment and we shall find that one case absolutely must have fixed work, and another have a removable bridge Some of these cases must be determined by careful study If we have a case in the anterior part of the mouth where it is easily kept clean there is no use to put on removable work, excepting to expedite repairs. On the other hand in teeth affected with pyorrhoea I think we should make a big mistake to put on removable work. If you have a case to supply that has a very short bite, such as supplying the first bicuspid in some close bite cases. I think this a case where we should use the fixed bridge. Where we have good firm abutments in the back part of the mouth, and the patient is not taking the care of the teeth that he ought, or the bite is heavy, this is an excellent place for the removable bridge.

Dr. Buttrick: There is one point in Dr. Ward's remarks with which I differ, that is as to the use of arsenic. I use cocaine almost exclusively. Once in a while I use the arsenic. I have had excellent results from the use of formaldehyde, a weak solution sealed into the tooth will dry up the pulp tissue fragments and in a few days you can be safe to pass a broach down to the apex of the root, and if you happen to leave a little, it is easily removed.

Dr. Ward: I feel the two agents are to be used with a good deal of judgment. I prefer to use the arsenic, but I also use the cocaine many times to get into the molars, and then leave the canal open long enough for the pulp tissue to break down. In regard to dessicating the pulp, we have several things that will accomplish this, but you can also use some agent that will cauterize and disintegrate the pulp tissue and get it out just as quickly as you would dry it up. The reference I made to cocaine was in regard to extracting the pulp and immediate root fiilling. Any one makes a mistake to think that he can sever the pulp in the upper molar anywhere near the apex.

A Member: It is not always necessary for us to make a permanent choice between removable and fixed bridges both of them having their place. I believe the same thing is true with the use of methods of devitalization of the pulp. If we have a pulp presented with much exposure and we know the pulp is in healthy condition, and we believe we can get to the end of those roots, then there is no doubt at all but what cocaine is the thing for us to use, but many of us find cases where we know there must be some remaining pulp in the canal. It may be full of germs and if you apply pressure you are liable to have trouble in the apical region, that is the place for arsenic. I have tried a number of times sealing in the paste but find it very irritating. For instance, the body of the pulp in the pulp chamber is perhaps already much disintegrated. In a case like that the patient would have trouble if we forced in the cocaine. We are liable to force it through the end of the root and cause trouble. I read one of our authorities, who said a man ought to have his sign taken down if he put arsenic into the root canals of any tooth. I do not agree with him in that statement. I believe if it is treated properly it can be done. We simply make trouble if we force a cocaine solution into the pulp where we know there is a septic condition.

Dr. Buttrick: In regard to what the last speaker has said, I would say there should be no question at all in regard to putting pressure on the pulp when it is partially disintegrated.

In a case like that I would clean out the necrotic tissue and seal in something that would disinfect; then at the next visit you will take out those pulp shreds without the slightest discomfort to the patient. Once in a while you will meet a tooth in which the pulp is exposed and you cannot touch it at all. I have sealed in a formaldehyde solution, and taken the pulp out without any trouble at all. I see no objection to letting the pulp break down by giving it a few days time.

Dr. Thompson: Dr. Ward showed me some bridge-work he had made, and everything that I have ever attempted myself was so inferior that I was quite discouraged. Those of you who have not seen it ought to get busy and see it. The paper seems to be a sort of a balm for wounded feelings, because if these gentlemen fail, it makes us feel good to see that they fall down where we ourselves do. It reminds me of an incident of a nephew, who said to an old uncle who was a very prosperous farmer.

"Uncle, with all these cows and this stream of water running through the place, you ought to sell a good deal of milk."

The Uncle jumped down out of the wagon and, shaking his fist at the youth, exclaimed: "I could meet every gallon of milk I have ever sold, in heaven, this minute."

Dr. Wood: I saw one bridge that Dr. Logan made that made me think a little and which was certainly next to perfection. I did not see his preparation. I have not seen any of Dr. Ward's work as yet, but I am still doing a little bridge-work of my own.

The statement of the difficulties in connection with crown and bridge work were well put. Since I have given considerable attention to prophylaxis, which a great many of those present also have done, my ideas of bridge-work have changed not entirely, but very greatly. I have never done bridge-work so that it looked like a piece of jewelry; I have never considered myself an expert gold worker, but have attempted to make a fit of the band of gold around the tooth but have failed in some cases, and have done some others successfully. After working along the line of prophylaxis and seeing so many of these cases where the bands were fitted perfectly and where the peridental membrane became very much inflamed in consequence of that band, so I have tried everything I could to avoid the band entirely. I have used the gold inlay for abutments, with the pins set into the root canals through the inlay, and have made flush joints of the band on the root, and have made crowns without any band at all to avoid the lap. No matter how perfectly the band laps the circumference of the root, it is impossible to avoid that irritation and consequent more or less inflammation at that point. I believe that fully as many bridges and abutments have been lost from the inflammation caused by that band as have been lost from faulty articulation. In times past the band has been driven up under the gum with a mallet never thinking of the peridental membrane. I want to add my caution to this practice because I feel the greatest importance of avoiding that band entirely in every case that we can.

So far as removable bridge-work is concerned, I hope some day to be able to at least decide when a removable bridge is preferable to a fixed bridge, and if I cannot do it myself to send it to somebody who can. So far I do not feel competent to do that class of work. I have always tried to avoid a lateral pressure on the bridge, in case of anterior teeth trying to make a square bite in every case if possible.

In regard to deciding between devitalization and not, devitalization, our dear friend Dr. Young put on a tooth for me and used a cataphoretic apparatus to grind the tooth down. He cut it so short that I thought he was not going to have any tooth left. I did not think he would make the crown stay on when he got it cut off; but I did not feel it. He cauterized it thoroughly with nitrate of silver and put on a dummy. I had a slightly sore tooth for nearly a month. That was some five years ago and it is in perfect condition today, a live healthy tooth. I have put on a good many since then myself with more or less success. I have asked several dentists what they had seen in the way of trouble with teeth crowned with live pulps, and fitted with gold crowns. I asked Dr. Watkins, and as he is a man that gives these things serious attention, he said that he had concluded that trouble came because you shut away the moisture from the crown of the tooth. I thought that was a very sensible conclusion. If we do any grinding and put a cap over the live pulp we do shut off that little moisture and dry that tooth out in a sense.

DR. LAND: The majority of practitioners do not have the time to consider the numerous points involved in good crown and bridge-work. Even at a meeting of this kind, if I should undertake to show my failures, the pathogical condiions, that we have to contend with, the fee that we get, all have a bearing on whether we ought to put in a bridge or not. If you will look back to the ancient times, 400 years before Christ, you will have the same evidence of the effect of bands and bridge-work between the incisors and cuspids, just as we have today, only a difference in the material used. There are times when nothing else will do; there are times when the open faced cap is the best thing to use and the only thing to use. There has not been a thing mentioned here tonight but what there are times when we ought to use it, but not always any one process. The paper read by Dr. Logan, to me is a very valuable paper, and what we lack is the time to thoroughly discuss it. Dr. Ward certainly has been conservative in his treatments and that is what we need. He spoke about some things that we failed in. That is all right. It is easy enough to fail in such intricate work.

I want to say to you that there are many bridges that I insert that I would not gaurantee 90 days and I have put them in under protest. It is only a short time ago that I fixed up a whole lot of loose teeth just like they would do 400 years before Christ, only I used a different kind of cement.

In regard to the technicalities of teaching the work so that the average young man would know how to do it. It is a remarkably intricate and expensive class of work and there are only the wealthier people who can afford to have it, and in the short length of time devoted to college work it cannot be taught except in an exclusive school, he should take the work here for such length of time as to develop skill and talent. We must charge large fees for bridge work, and in proportion as these fees are exacted we have that much more responsibility.

I have used arsenic for over forty years, and have never used cocaine; never found any use for it. I will take the same tooth that you use cocaine on, and use nothing but pure carbolic acid. If I cannot expose that nerve, and then take a little bit of cotton wet with pure carbolic acid and put it over the pulp and take a fine instrument and gently tap that to get enough pressure to strangle the pulp, and then you can get it out without any cocaine; you strangle it by pressure. Dr. Wood speaks about working above the gum line. You think because you put a band beneath the gum line, you have done something, because you have hid the joint, I should say, get it up so that you can see it. When Dr. Richmond came to this city many years ago and gave instruction in putting on Richmond crowns I put one on and lost the tooth from peridental inflammation. It was a central incisor on which I put on the band, and have never made one since and hope I never shall.

So far as covering up teeth is concerned, so that they do not have any chance to "breathe" I have got one that has been in sixteen years and have never had any trouble yet. When I found that they were irritating the gum I commenced to take them off and put on metal caps, I found the cement under them in healthy condition. The cement does not injure the pulp unless it has excessive acid in it. I have taken off hundreds of these caps and found them just as sensitive as the day I put them on.

Dr. Ward: The life of the pulp in bridge-work means more to me than any other one thing. I was taught never to destroy a pulp that could be saved under any conditions and I am practicing it today, but the more bridge-work I do and oftener I need to do it. We cannot prepare all roots the way they ought to be with a live pulp. I do not care, if I am going to crown or band the root whether the pulp is dead or not, but when I expect to fill a tooth it is different, I prefer to save the pulp. I would like to make that distinction between saving or devitalizing the pulp, whether I am going to band it for a bridge or to fill it. In the case of inlays and crowns for bridge work because of extensive cutting for anchorage we must devitalize.

Dr. Buttrick: There is one point that has not been mentioned at all, the use of gutta percha cement for setting fixed bridge work; it is easy to get just the right amount by adding or subtracting a little, and when you want to get it off you can take it off very readily by warming your abutments.

Dr. E. D. Spaulding: In listening to the discussion I am reminded oi David Harum's expression, that "a certain amount of fleas is a good thing for a dog, to make him forget that he is a dog." If we remember our failures and piofit by them it is a good thing that we do not get too "chesty".

I do not think there is any subject in which we are instructed to the extent that we should be in college, there isn't time and students as a rule, could not assimilate it all if the instruction was given. It is up to the practitioner when he gets out to learn considerably more, and I think that the only way we can do that is by reading our Journals and attending the society meetings. I am sure that we shall get a new stimulus and learn something about bridge-work by listening to Dr. Logan's paper and the discussion tonight.

In reference to attaching bridge-work to pyorrhoea teeth, to which Dr. Logan objected, I think there are a good many cases where the attachment of a certain kind of bridge to a tooth affected by pyorrhoea, supposing we are going to treat that tooth, that will assist materially in prolonging the life of that tooth, rather than to let it stand alone and receive dislocating impacts or motions without the aid of attachment to some other teeth. I think in the majority of cases the teeth are getting excessive lateral motion. If it is the bucco-lingual motion by its being tied to another tooth it received some assistance and it is not continually irritated by forces of mastication. These teeth are usually strong enough to withstand the pressure in direct line with the root. A patient came to me in 1894 who had a lower right second molar that was quite badly affected with pyorrhea. The first molar was out, also the two bicuspids. The second molar had moved forward so that it was impossible to put in but two bicuspids. He asked me to put in a bridge, and I objected saying that it would not last. I said it might last for a short time, but not long. He said if it will last two years I am willing to pay for it. I had to repair it two years ago but he is still wearing a bridge upon these identical teeth, and the molar is in better condition than the day I put on the bridge, the reason is that it has been tied to another tooth and it did not have the motion incident to mastication.

Dr. Watkins: I made a bridge from the cuspids to the third molars, the third molars on each side were almost out. At the palatine root you could slip a small probe nearly to the end of the root. The party insisted upon having bridges; she came three times and I refused each time thinking it a hopeless case. The fourth time I gave in on condition that she should take the entire responsibility of the case; if it did not last her a week that was altogether her fault. Under these conditions I made it, and that was ten years ago. The teeth today appear better than they did then; they seem more solid, and it is doing good service now and is more solid than in the first place. So I do not know where the limit is as to where we have to stop in putting on a bridge. There is hardly any limit to it.

Dr. Land: Some teeth that I have covered that way and would not recommend them six months I have known to last often 15 years. I had a bad case in which the teeth became tight after they were bridged; this patient died of heart disease fifteen years afterwards.

Dr. Logan: From Dr. Ward's criticism I judge he does not favor amputation of the roots. I amputated the root of an upper second molar, about 1902, as I remember. That tooth today forms the posterior abutment of a bridge and there are twelve teeth to the bridge. You must, of course, have your patient take care of these teeth. I maintain that amputation is practical and permissable and that you will often gain excellent results. If the tooth was to form the posterior abutment of the bridge from the second molar to the cuspid only then you have a difficult condition; but supposing you should carry that around to the cuspid on the opposite side of the mouth, I claim that you can get excellent results from a case of that kind. Such construction ties your teeth in place and prevents that lateral movement.

I cite another case where the bridge is anchored to the second molar and the first bicuspid that has been in five years, and it is giving excellent results.

It is the root preparation that counts in every instance. The mode of using amalgam and gold fillings for retaining bridges are, as I have stated, all right in cases where your occlusion is slight or wanting, but if there is any unusual strain brought to bear upon the dummies that are placed in this manner it is a matter of a very short time before your filling or tooth substance is bound to weaken under the strain. On the other hand if your abutment is formed with the gold inlay at the base you have a different condition and you will obtain excellent results from such a method. Anchor any bridge through an amalgam filling to a single rooted tooth and you cannot expect good results because the movement of this tooth is such that it is bound to loosen your filling unless the occlusion is very slight.

Regarding cocaine for devitalizing, I do not pretend to be an authority upon the subject of devitalization. Dr. Wood was speaking of a tooth that had been treated by Dr. Young, when dentine was cauterized by silver nitrate. Dr. Hayes would remember in my junior year in Ann Arbor he was doing some crown work in the laboratory for my own mouth. He said: You are fixing up for a bad case of neuralgia some night by crowning those teeth with live pulps. That was 11 years ago. I have not lost any sleep on account of those teeth and they are just as vital as they were when the crowns were put on. You get the surface of the tooth protected so that it will tend to ward off any extraordinary emergency that might cause trouble afterwards.

I do not hesitate in any case to use silver nitrate in cauterizing the stumps of the roots if I am going to crown. I usually apply a rubber and blow the silver nitrate direct from the crystal; dip the crystal in water and smear it over the tooth. I have never succeeded in getting anything that would act on the dentine that would numb it to that extent so that I could reduce the tooth without distress to the patient. I think it is however, more than anything else, the result of friction. I think the smaller the surface that you come in contact with at a given time the better; keep the stone moving rapidly and well lubricated with warm water.

There is a question in my mind whether the use of the high pressure injection may not be detrimental to the life oı the pulp. Dr. Land spoke about inserting bridges under protest and about the funeral being held later on. I think we have all had experience in that line. I had a patient not many months ago who had only the upper second molars on either side. She wished a fixed bridge which I declined to make. It was inserted, but has been since removed and the patient is now wearing a plate to good advantage. No doubt the dentist who inserted that bridge did so under protest, but in this case the patient is out for his scalp. I think if a man cannot do conscientious work along the line of bridge work, he better not do it at all. Where a man is looking for the almighty dollar he will have more failures than if he does it from a conscientious standpoint.

Dr. Land spoke about making one Richmond crown and then discarded their use entirely. About two years ago I had the pleasure of seeing a mouth that Dr. Richmond himself had worked upon. The crown had been in twenty years and the tooth was perfectly firm as far as I could see. There was in the same mouth a bridge extending from the first molar to the cuspid, but there was no anterior attachment. This had been in 20 years and had done good service, the molar was, however, slightly loosened, I think that you get most desirable results sometimes by bridging the teeth affected with pyorrhoea and holding them in a rigid condition. I have had teeth that have been decidedly shaky and after they have been bridged for some time they have become firmer and the life of the teeth has been materially extended.

